# Impact of COVID-19 mitigations on anxiety and depression amongst university students: A systematic review and meta-analysis

**DOI:** 10.7189/jogh.13.06035

**Published:** 2023-09-01

**Authors:** Bohee Lee, Prerna Krishan, Lara Goodwin, Damilola Iduye, Emma Farfan de los Godos, Jodie Fryer, Kate Gallagher, Kaitlyn Hair, Eimear O'Connell, Kristen Ogarrio, Theresa King, Shifa Sarica, Eileen Scott, Xue Li, Peige Song, Marshall Dozier, Emilie McSwiggan, Kristefer Stojanovski, Evropi Theodoratou, Ruth McQuillan, UNCOVER group

**Affiliations:** 1 Usher Network for COVID-19 Evidence Reviews (UNCOVER) group, Usher Institute, University of Edinburgh, Edinburgh, UK; 2 Centre for Population Health Sciences, Usher Institute, University of Edinburgh, Edinburgh, UK; 3 National Heart and Lung Institute, Imperial College London, London, UK; 4 School of Public Health and Tropical Medicine, Tulane University, New Orleans, Louisiana, USA; 5 Public Health Scotland, Edinburgh, UK; 6 School of Public Health, Zhejiang University School of Medicine, Hangzhou, China; 7 Information Services, University of Edinburgh, Edinburgh, UK; 8 Advanced Care Research Centre, University of Edinburgh, Edinburgh, UK; 9 Centre for Global Health, Usher Institute, University of Edinburgh, Edinburgh, UK

## Abstract

**Background:**

While much research has addressed mental health concerns related to the coronavirus disease 2019 (COVID-19) pandemic, there remains a scarcity of studies specifically exploring the changes in anxiety and depression among university students before and after the implementation of COVID-19 mitigation measures.

**Methods:**

In this systematic review and meta-analysis, we searched databases including MEDLINE (Ovid), EMBASE (Ovid), PsycINFO (Ovid), CINAHL (EBSCO), ERIC (EBSCO), the WHO COVID-19 database, Scopus, and Science Citation Index (Web of Science) as of 15 February 2023. We included studies that used a validated tool to measure changes in anxiety or depression at two distinct time points – before (T1) and during (T2); during (T2) and after (T3); or before (T1) and after (T3) COVID-19 mitigation. The quality of studies was assessed using an adapted Joanna Briggs Institute Checklist for longitudinal studies. Utilising random-effects models, we synthesised changes in continuous outcomes as standardised mean difference (SMD) with 95% confidence interval (CI) and binary outcomes as risk difference (RD) with 95% CI.

**Results:**

In total, 15 studies were included in this review, with eight of moderate and seven of high quality. In most of the included studies (n = 13), the majority of participants were women. Eleven studies analysed mental health outcomes between T1 and T2 of COVID-19 mitigations. Continuous symptom changes were a minimal or small improvement for anxiety (SMD = -0.03, 95% CI = -0.24 to 0.19, *I^2^* = 90%); but worsened for depression (SMD = 0.26, 95% CI = -0.01 to 0.62). However, the proportions of students reporting moderate-to-severe symptoms, defined by specific cut-offs, increased during COVID-19 mitigation measures for both anxiety (RD = 0.17, 95% CI = -0.04 to 0.38, *I^2^* = 95%) and depression (RD = 0.12, 95% CI = 0.03 to 0.22, *I^2^* = 72%). Sensitivity analyses, which distinguished between baseline periods based on awareness of COVID-19, demonstrated an exacerbation of both symptoms when comparing the period before the global awareness of the COVID-19 outbreak (before December 2019) with the period during the implementation of mitigation measures.

**Conclusions:**

Mental health outcomes, especially depressive symptoms, were observed to worsen in university students during COVID-19 mitigations. Despite considerable heterogeneity requiring careful interpretation of results, the impact of COVID-19 mitigations on mental health in university students is evident.

**Registration:**

PROSPERO (CRD42021266889).

The coronavirus disease 2019 (COVID-19) pandemic placed significant stress on populations around the world, leading to severe consequences for mental health [1,2]. Some of these stressors, such as the fear of infection for self and loved ones, and uncertainty about the future, were associated directly with the virus. Others were linked to the extensive mitigation measures that governments across the world put in place, in an attempt to prevent or reduce virus transmission. Mitigation measures included social distancing rules, which curtailed in-person interaction; lockdowns, which isolated people in their own homes; and severe restrictions on travel. Whilst many studies investigated the overall mental health impacts of the pandemic overall, there is a paucity of evidence focusing specifically on the mental health impacts of COVID-19 mitigation measures.

One group particularly impacted by COVID-19 mitigation measures worldwide was students in post-secondary education. Students experienced disruption to their education and isolation from their normal social networks during the pandemic. At short notice, most were required to leave campus. Those who remained (for example, overseas students unable to return home) were locked down in their student accommodation. Face-to-face teaching, educational, sporting and social events and activities on campus were abruptly cancelled. Additionally, teaching and pastoral support services quickly moved online. The scale of these impacts was unprecedented: a monitoring study by UNESCO found that over 100 countries implemented nationwide closures of educational institutions during the early phase of the pandemic, with localised closures in several more countries [3]. Students may be particularly vulnerable to adverse mental health outcomes due to the profound disruptions to normal life engendered by COVID-19 mitigation measures. Young people in their late teens and early twenties undergo a developmentally significant transition from adolescence to early adulthood [4]. During this crucial period, the developing brain is particularly sensitive to environmental stressors [5]. This is also when many mental illnesses, including anxiety and depression, often first manifest themselves [5]. The purpose of this review was therefore to assess the impact of COVID-19 mitigation strategies, such as lockdowns and the rapid transition to online learning, on the prevalence of anxiety and depression among post-secondary school students.

## METHODS

This systematic review is registered on PROSPERO (CRD42021266889). We followed the Preferred Reporting Items for Systematic Review and Meta-Analysis (PRISMA) reporting statement [6].

### Search methods

Our search strategy combined four primary themes: COVID-19, anxiety or depression, university students, and COVID-19 mitigations. We utilised Boolean operators to effectively search the MEDLINE (Ovid), EMBASE (Ovid), CINAHL (EBSCO), PsycINFO (Ovid), ERIC (EBSCO), WHO COVID-19 database, Scopus, and Science Citation Index (Web of Science) databases on 15 February 2023. Additionally, we conducted forward / backward citation tracking of included studies to capture any relevant articles missed in the searches. Detailed search strategies are available in Table S1 in the **Online Supplementary Document**. We used the Systematic Review Accelerator (https://sr-accelerator.com/#/deduplicator) software to remove duplicates. After deduplication, the resulting records were imported into Covidence, a systematic review software (https://www.covidence.org/).

### Screening and selection of studies

Two independent reviewers (LG, DI, KH, BL, JF, PK, EF, EO, KO, KG) initially screened the titles and abstracts of the retrieved studies. This was followed by a full-text screening to identify eligible studies. As our aim was to compare changes in anxiety / depression within the same subjects at different time points, we established definitions and key decisions based on the eligibility criteria (**Table 1**). During the title, abstract, and full-text screening, we included studies that assessed the impact of any COVID-19 mitigation measures, implemented by either the government or educational institutions, on anxiety / depression in the same participants at a minimum of two different time points. These points could be before (T1), during (T2), or after (T3) the implementation of the COVID-19 mitigation measures. However, we excluded studies that did not explicitly mention any COVID-19 mitigation measures or those that compared different participants. We did not have any language or geographical region limitations to allow a complete global search and avoid potential bias. Any disagreements were resolved through discussion or by involving a third independent reviewer.

**Table 1:** Eligibility criteria for selecting studies

	Inclusion	Exclusion
Population	Post-secondary student populations	Non-post-secondary school students
Exposure	Any COVID-19 mitigation implemented by the government or educational institution (country or institutional level)	Studies without a COVID-19 mitigation or those which only referenced the “pandemic” broadly
Comparator	At least two data collection measurements: pre, post and during the COVID-19 mitigation within the same cohort	Only one data collection measurement; studies compared different cohorts
Outcome	Point estimates or the number of clinically significant cases, measured using a validated measurement tool	Other mental health outcomes including well-being, sleep quality, or suicidal ideation; outcomes measured using non-validated measurement tools
Setting	Studies with college, undergraduate, and postgraduate students in post-secondary educational institutions as participants	Studies involving participants in residency training programmes; high school pupils attending college for additional qualifications; students on work placements, internships, apprenticeships, and graduate schemes
Study design	Empirical studies with within-cohort comparisons	Empirical studies with between-cohort comparisons, modelling studies, opinions, editorials, reviews, and conference abstracts
Geographical location	Studies conducted in any country or countries	No restrictions based on geographical location

### Data extraction and quality assessment

Prior to formal data extraction, we piloted a data extraction form using three randomly selected studies. Two independent reviewers then extracted the following data: study design, setting, demographic information, data collection dates, and types of COVID-19 mitigation measures. If a study measured anxiety /depression at more than two-time points before the mitigations, we extracted all data from different time points. However, when multiple measurements were taken following the initiation of mitigation actions (e.g., before lockdown, first lockdown, and second lockdown), we only extracted data from the first mitigation (e.g., first lockdown) to match with the baseline outcome.

We utilised the Joanna Briggs Institute (JBI) checklist for longitudinal studies to assess the quality of evidence in the included studies [7]. Given that these studies examined changes in anxiety / depression within the same subjects over time, it was deemed appropriate to classify them as longitudinal studies. Such design enabled quantifying changes in mental health outcomes in the context of COVID-19 mitigation measures. Two independent reviewers (LG, DI, KH, BL, JF, PK, EF, EM, EO, KO, KG) carried out quality assessments for each study, with ratings classified as either “high”, “moderate”, or “low” quality. Any disagreements or discrepancies were reconciled through discussion, or, if necessary, decided by a third reviewer.

### Data synthesis and data analysis

We synthesised the data using either narrative synthesis or meta-analysis. We employed narrative synthesis for studies that did not provide exact point estimates, or those we deemed highly heterogeneous compared to others. For continuous outcomes, we extracted the means and standard deviations (SD) of outcomes from two different time points and provided the standardised mean difference (SMD) along with 95% confidence intervals (CIs) as summary estimates to describe a change in terms of within-group SD rather than raw change scores. Moreover, for studies that examined proportions of clinically significant anxiety or depression – defined as the number of participants above a cut-off point on a measurement scoring tool – we calculated risk differences (RD) with 95% CIs to examine the change in proportions between two time points. We employed residual maximum likelihood (REML) in the inverse variance weighted random-effects meta-analysis.

We hypothesised that baseline mental health outcomes could potentially influence the observed changes in these outcomes. To test this, we classified the baseline time points (T1) into three categories based on the global awareness of COVID-19: T1a (before the global COVID-19 outbreak, before December 2019), T1b (after the outbreak but before the pandemic, in December 2019), and T1c (during COVID-19 but before mitigations, from January 2020 onwards). Consequently, in a sensitivity analysis, we evaluated these changes using the various baseline time points. In addition, we did a subgroup analysis to evaluate the impact of different COVID-19 mitigations on mental health outcomes.

We did not evaluate potential “small-study effects” using funnel plots for visual assessment or Egger’s test, as the power of these tests would be insufficient to distinguish chance from real asymmetry due to the small number of studies included in each meta-analysis (fewer than 10 studies). Heterogeneity among individual studies was assessed using *tau^2^* and *I^2^* metrics. We set the threshold for statistical significance at 5%. All meta-analyses were conducted in R (version 4.1.2) using the meta package.

## RESULTS

**Figure 1:**
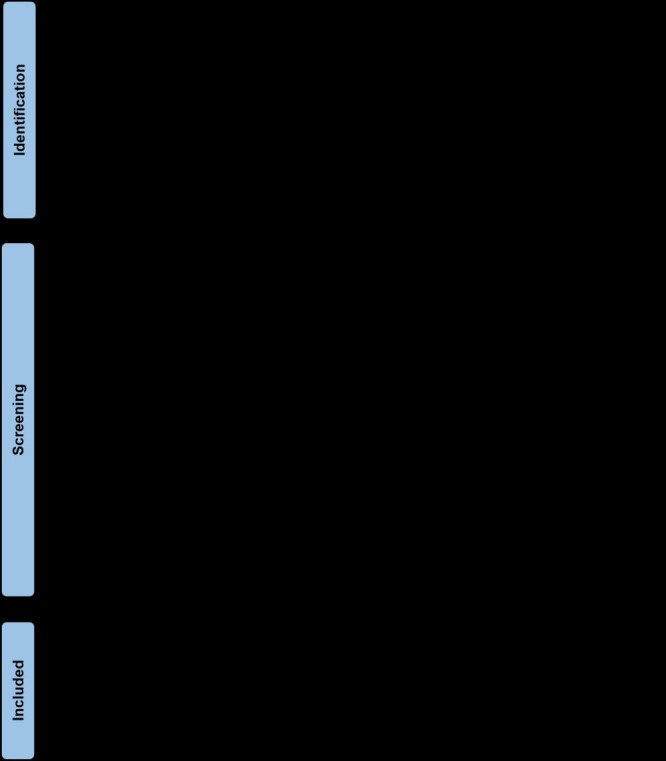
Preferred Reporting Items for Systematic Reviews and Meta-Analyses (PRISMA) flow diagram.

Our search yielded 3951 unique records after deduplication. After title and abstract screening, 87 remained. After the full-text screening, 15 were retained for the final analysis [8-22]. Search results are summarised in a PRISMA diagram (**Figure 1**).

### Characteristics of studies

**Table 2** summarises the characteristics of the included studies. All 15 studies were longitudinal studies, comparing anxiety / depression levels between different time points within the same cohort of students. The study size varied from 40 in a study from Brazil [20] to 35 516 students in a study from China [19] (median (mdn) = 195, interquartile range (IQR) = 328). In 12 of the 16 studies, the samples were heavily skewed towards female participants (range = 58.5-86%). Male students were dominant in two studies [8,19]. There were three studies from the USA [10,12,15], four from China [9,16,18,19], two from Germany [17,22], one each from France [11], Portugal [13], Switzerland [8], Brazil [20], New Zealand [21] and the UK [14].

**Table 2:** Study and demographic information of the included studies (n = 15)

Author (year)	Study design	Country	COVID-19 mitigation measures	Data collection timing related to COVID-19 mitigation measures			N of students (% of female)	Outcome(s)
				**T1**	**T2§**	**T3‖**		
Elmer 2020 [8]	Longitudinal	Switzerland	Lockdown (Apr 2020)	T1a*: Sep 2019	Apr 2020	NA	142 (15.3%)	Anxiety, depression
Li 2020 [9	Longitudinal	China	Lockdown (Jan 2020)	T1b†: 20 Dec 2019	Feb 2020	NA	555 (76.8%)	Anxiety, depression
Zhang 2020 [10]	Longitudinal	USA	Lockdown & online learning (Mar 7, 2020)	T1c‡: 1 Jan to 29 Feb 2020	Mar 29 to 31 May 2020	NA	49 (61.2%)	Anxiety, depression
Charbonnier 2021 [11]	Longitudinal	France	Lockdown (April 23 and Nov 20, 2020)	NA	23 Apr to 8 May 2020 (first lockdown)	9 to 23 Jun 2020 (after first lockdown)	91 (73.6%)	Anxiety, depression
Coughenour 2021 [12]	Longitudinal	USA	Lockdown (Mar 31, 2020)	T1c‡: 17 to 31 Mar to 2020	7 to 28 May 7 2020	NA	194 (73%)	Depression
Conceição 2021 [13]	Longitudinal	Portugal	Lockdown (Jun 2020 and Mar 2021)	T1a*: Oct 2019	Jun 2020 (1^st ^lockdown) Mar 2021 (2^nd^ lockdown)	NA	366 (71.3%)	Anxiety, depression
Evans 2021 [14]	Longitudinal	UK	Lockdown (Apr / May 2020)	T1a*: Oct 2019	1 Apr to 30 May 2020	NA	254 (86%)	Anxiety, depression
Fruehwirth 2021 [15]	Longitudinal	USA	Lockdown (Late Mar 2020)	T1b†: Oct 2019 to Feb 2020	NA	Jun to Jul 2020	419 (70.4%)	Anxiety, depression
Lu 2021 [16]	Longitudinal	China	Lockdown (Jan 23-25, 2020)	T1a*: 16 Sep to 23 Oct 2019	NA	16 to 23 Apr 2020	5181 (62.2%)	Anxiety, depression
Schindler 2021 [17]	Longitudinal	Germany	Online learning (Jun 2020)	T1a*: Oct 2019 T1b†: Dec 2019 (1^st^ semester)	Jun (2^nd^ semester) Dec 2020 (3^rd^ semester)	NA	63 (63.5%)	Depression
Yang 2021 [18]	Longitudinal	China	Lockdown (Jun 2020)	T1a*: Dec 2018 T1a*: Jun 2019 T1b†: Dec 2019	Jun 2020	NA	195 (58.5%)	Depression
Huang 2022 [19]	Longitudinal	China	Lockdown (Jan to Mar 2020)	NA	3 to 10 Feb 2020 (remission period 24 Mar to 3 Apr 2020)	1 to 15 Jun 2020	35 516 (26.0%)	Depression
Seffrin 2022 [20]	Longitudinal	Brazil	Online learning (Jul 6, 2020)	T1c‡: 22 to 25 Jun 2020 (no online or presential): end of presential class: 16 March 2020	15 to 19 Aug 2020	NA	40 (75.0%)	Anxiety, depression
Slykerman 2022 [21]	Longitudinal	New Zealand	Online learning (Mar 25, 2020)	T1c‡: Mar 2020	Nov 2020	NA	391 (NA)	Anxiety
Weber 2022 [22]	Longitudinal	Germany	Second lockdown (November 2020)	T1c‡: 20 Jul to 28 Aug 2020	10 Nov to 2 Dec 2020	NA	135 (74.1%)	Anxiety, depression

NA – not available

*Before COVID-19 measures implementation and before COVID-19 pandemic.

†Before COVID-19 measures implementation and in early stage of COVID-19 outbreak.

‡Before COVID-19 measures implementation and after COVID-19 pandemic.

§During COVID-19 measures implementation.

‖After COVID-19 measures implementation.

### Timing of data collection and measurements

The timing of data collection in the studies was determined by mitigation measures, including a combination of societal mitigations and context-specific mitigations. Societal mitigations are variously described as lockdown, quarantine, stay-at-home orders, home confinement, curfew, bans on non-essential movement outside the home, prohibition or restriction of social gatherings, the closure of essential businesses and public spaces and social distancing. Context-specific mitigations are variously described as campus closure, closure of student housing, sending students home, relocation from campus, online learning and home learning. The timing and intensity of these measures differed across countries and time.

Eleven studies analysed mental health outcomes between T1 and T2 during COVID-19. Baseline anxiety / depression levels were measured before the COVID-19 outbreak (T1a) in six studies [8,13,14,16-18]; after the outbreak but before the pandemic (T1b) in four studies [9,15,17,18]; and after pandemic but before mitigations in five studies [10,12,20-22]. Two studies had at least two baseline time points for anxiety / depression [17,18].

In three other studies [17,20,21], the main mitigation measures were the transition to online learning due to university closures. Two studies that compared data from T1 and T3 focused on lockdown [15] or stay-at-home orders [16]. Two others conducted surveys between T2 and T3 [11, 19]. Most surveys were conducted between April and July 2020 during the pandemic.

One study conducted four measurements: during France’s first national lockdown, during the period after the lockdown with university closure and summer vacation, when universities were reopened, and during the second national lockdown [11]. In our analyses, we only included the periods before and after the first lockdown, as we judged that this would provide a clearer comparison of changes in mental health outcomes. Huang et al. (2022) measured mental health outcomes during three different periods: the lockdown phase (during nationwide control measures), the remission phase (the period when infection rates were under control), and the normal prevention phase (no longer nationwide control but local or individual level infection prevention measures, coinciding with the reopening of universities) [19]. To explicitly assess the impact of the lockdown, we included only the levels of mental health outcomes during the lockdown period and the normal prevention period.

### Outcome measures

Most of the studies (n = 10) examined both anxiety and depression. However, four studies [12,17-19] focused solely on depression, and one study exclusively examined anxiety. Seven of the studies [11,13-16,20,22] reported on the prevalence of moderate-to-severe depression between two-time points, while eight studies [11,13-16,19,20,22] reported on the prevalence of moderate-to-severe anxiety.

Various scales were used to assess anxiety and depression. For anxiety, the General Anxiety Disorder scale (GAD-7) [23] was the most used tool, employed in seven studies [8,10,13,15,16,20,22]. For depression, the Patient Health Questionnaire (PHQ)-9 [24] was frequently used, appearing in seven studies [10,12,13,16,17,20,21]. Three studies used a single tool to measure both anxiety and depression: the PHQ-4 [25] was used in one study [9] and the Hospital Anxiety and Depression Scale (HADS) [26] was used in two studies [11,14].

The cutoffs to define clinically significant or moderate-to-severe anxiety / depression are summarised in Table S3 in the **Online Supplementary Document**. Four studies [13,14,20,22] reported the prevalence of moderate to severe anxiety (defined as GAD-7 ≥ 10 [13,20,22], HADS≥8 [14]). Meanwhile, five studies reported the prevalence of depression, defined as PHQ-9 ≥ 10 [13,20], PHQ-8 ≥ 10 [22], HADS≥8 [14] and CES-D≥16 [18]).

### Study quality

The results of quality assessments are shown in Table S2 in the **Online Supplementary Document**. Of the 15 studies evaluated, eight were assessed as moderate quality [11-13,16,17,19,20,22]. The remaining studies were assessed as high quality [8-10,14,15,18,21].

### Change in anxiety / depression between different time points

**Figure 2:**
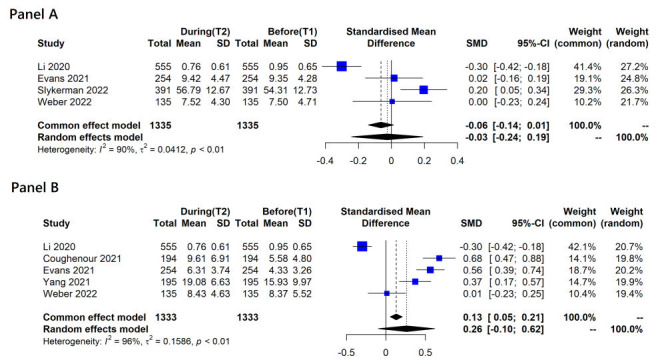
Overall change in continuous anxiety or depression score between different time points. **Panel A**. Anxiety. **Panel B**. Depression.

The forest plots (**Figure 2**, panel A and panel B) illustrate the SMD for anxiety / depression symptoms in studies of university students. In terms of the period before and during mitigation measures (T1 and T2), the estimated change in anxiety in four studies [9,14,21,22] was not significant and approximated zero (SMD = -0.03, 95% CI = -0.24 to 0.19, *I^2^* = 90%, *tau^2^* = 0.041). However, an increased level of depression during mitigations was observed in five studies (SMD = 0.26, 95% CI = -0.01 to 0.62, *I^2^* = 96%, *tau^2^* = 0.159), although there was no statistical significance in the meta-analysis [9,12,14,18,22]. Li et al. (2020) measured anxiety / depression in December 2019 (T1b) and reported that the direction of change in anxiety and depression was entirely opposite [9]. In this study, the mean score of baseline anxiety / depression (before mass confinement) was higher than after confinement, which was attributed to fears of infection or concerns about academic progress.

In a sensitivity analysis including studies that conducted surveys at T1a, two studies were available, but they could not be included in a meta-analysis due to different statistical metrics [8,14]. More anxious and depressive symptoms were observed during mitigation measures compared to baseline (T1a) in a study by Evans et al. (2021). However, the increase was statistically significant only for depressive symptoms, not for anxiety (mean difference (MD) = 0.072, *P* = 0.782 for anxiety and MD = 1972, *P* < 0.001 for depression). Similarly, in a study by Elmer et al. (2020), the students reported being more depressed (MD = 4.44, standard error (SE) = 0.50, *P* < 0.001), and slightly more anxious compared to baseline (T1a) (MD = 0.60, SE = 0.24, *P* = 0.014). However, when including studies conducting surveys exclusively during the pandemic (T1c), the level of anxiety worsened, though not significantly (SMD = 0.13, 95% CI = -0.05 to 0.31, *I^2^* = 45%, *tau^2^* = 0.008) [21,22]. Conversely, the level of depression significantly worsened during the mitigation in two studies with an SMD of 0.58 (95% CI = 0.44-0.71, *I^2^* = 0%, *tau^2^* = 0) [14,18].

Fruehwirth et al. [15] investigated continuous changes in anxiety / depression before and after (T1b and T3) mitigation measures. They reported a minimal or slight increase in anxiety and a slight increase in depression after lifting the lockdown (MD = 0.072, *P* < 0.01 for anxiety and MD = 0.103, *P* < 0.001 for depression, respectively) [15].

### Change in prevalence of moderate-to-severe anxiety / depression

**Figure 3:**
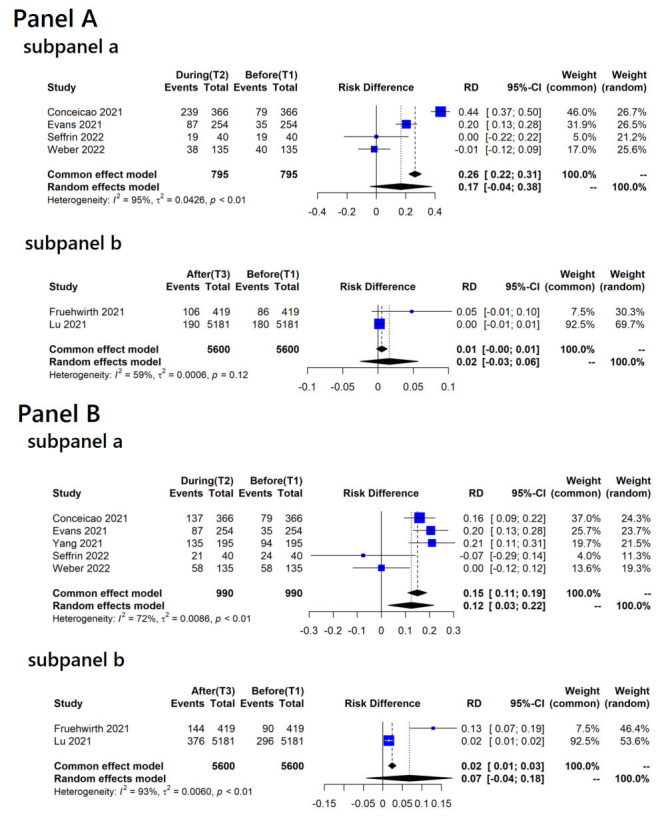
Risk differences in proportions of participants reporting moderate-to-severe anxiety or depression score between different time points. **Panel A**. Change in proportions of participants reporting moderate-to-severe anxiety. Subpanel a: change in proportions of moderate-to-severe anxiety between before (T1) and during (T2) COVID-19 mitigation measures; subpanel b: change in proportions of moderate-to-severe anxiety between before (T1) and after (T3) COVID-19 mitigation measures. **Panel B**. Change in proportions of participants reporting moderate-to-severe depression. Subpanel a: change in proportions of moderate-to-severe depression between before (T1) and during (T2) COVID-19 mitigation measures; subpanel b: change in proportions of moderate-to-severe depression between before (T1) and after (T3) COVID-19 mitigation measures.

**Figure 3** illustrates changes in the proportions of participants reporting moderate-to-severe anxiety / depression across different time points. Overall, the pooled differences indicated an increase in the proportions of moderate-to-severe anxiety during mitigations (**Figure 3**, panel A, subpanel a), along with a significant increase in moderate-to-severe depression (RD = 0.17, 95% CI = -0.04 to 0.38, *I^2^* = 95%, *tau^2^* = 0.043 for moderate-to-severe anxiety and RD = 0.12, 95% CI = 0.03-0.22, *I^2^* = 72%, *tau^2^* = 0.009 for moderate-to-severe depression, respectively) (**Figure 3**, panel B, subpanel a). However, when comparing the proportions of moderate-to-severe anxiety / depression before and after mitigations, no significant changes were observed, with values close to zero (RD = 0.02, 95% CI = -0.03 to 0.06, *I^2^* = 59%, *tau^2^*<0.001 for moderate-to-severe anxiety and RD = 0.07, 95% CI = -0.04 to 0.18, *I^2^* = 93%, *tau^2^* = 0.006 for moderate-to-severe depression, respectively) (**Figure 3**, panel A, subpanel b and panel B, subpanel b).

In a sensitivity analysis including studies comparing baselines during the COVID-19 pandemic (T1c only) and during mitigations, there was no change in moderate-to-severe anxiety with an RD of -0.01 (95% CI = -0.11 to 0.09, *I^2^* = 0%, *tau^2^* = 0) in two studies [20,22]. Conversely, when including studies comparing baselines before the COVID-19 outbreak (T1a only) and during mitigations, there was a significant increase in proportions of participants reporting moderate-to-severe anxiety with an RD of 0.32 (95% CI = 0.09-0.55, *I^2^* = 95.5%, *tau^2^* = 0.026) [13,14]. In the context of moderate-to-severe depression, a separate sensitivity analysis for T1c and during mitigations showed an RD of -0.02 (95% CI = -0.12 to 0.09, *I^2^* = 0%, *tau^2^* = 0) in two studies [20,22]. For change between T1a and during mitigations, there was a significantly increased proportion of moderate-to-severe depression (RD = 0.21, 95% CI = 0.14-0.27, *I^2^* = 51.4%, *tau^2^* = 0.002) [13,14,18].

### Impacts of COVID-19 mitigation measures

#### Lockdown

**Figure 4:**
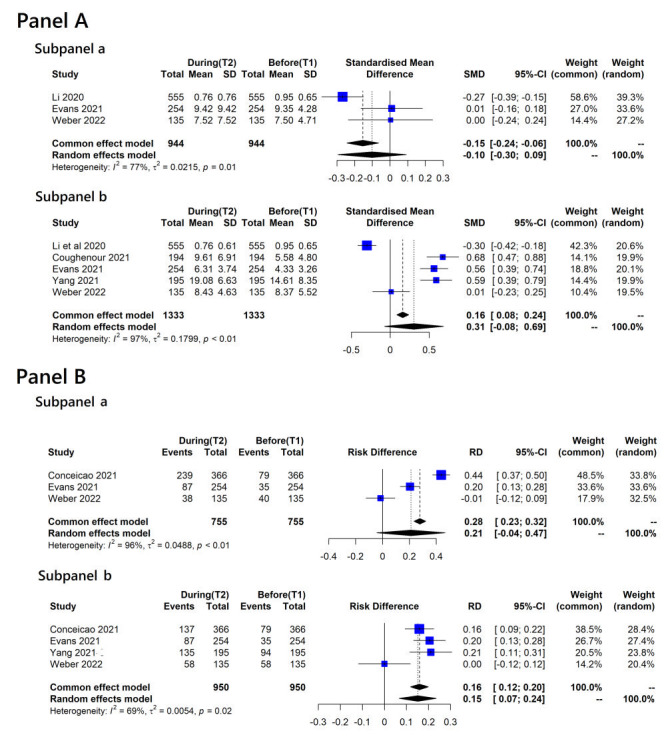
Change in anxiety and depression during the lockdown. **Panel A**. Change in continuous outcomes. Subpanel a: anxiety; subpanel b: depression between before (T1) and during (T2) the lockdown. **Panel B**. Change in proportions of participants reporting moderate-to-severe. Subpanel a: anxiety; subpanel b: depression between before (T1) and during (T2) the lockdown.

Nine studies reported changes in anxiety and depression during lockdown [8-10,12-15,18,22] (**Figure 4**). For a continuous change in mental health outcomes (**Figure 4**, panel A, subpanel a and subpanel b), the meta-analysis revealed a minimal or slight improvement in anxiety across three studies comparing periods T1 and T2 (SMD = -0.10, 95% CI = -0.30 to 0.09, *I^2^* = 76.9%, *tau^2^* = 0.022) [9,14,22]. However, we observed worsened depression during the lockdown in five studies (SMD = 0.31, 95% CI = -0.08 to 0.69, *I^2^* = 96.7%, *tau^2^* = 0.180) [9,12,14,18,22].

When considering only baselines before the COVID-19 outbreak (T1a) and during lockdowns, continuous depression outcomes significantly worsened (SMD = 0.58, 95% CI = 0.44-0.71, *I^2^* = 0.0%, *tau^2^* = 0) in two studies [14,18]. Only one study was available for anxiety, which reported a minimal change during lockdowns (MD = 0.072, *P* = 0.782) [14]. When including studies with baselines at T1b or T1c, the continuous outcome of anxiety improved (SMD = -0.15, 95% CI = -0.42 to 0.11, *I^2^* = 75.0%, *tau^2^* = 0.028) in two studies [9,22], whereas the continuous outcome for depression worsened (SMD = 0.13, 95% CI = -0.44 to 0.68, *I^2^* = 97.0%, *tau^2^* = 0.242) in three studies [9,12,22] during the lockdown.

Concerning the changes in proportions of reported moderate-to-severe anxiety and depression, the meta-analyses showed a higher prevalence of both mental health outcomes during lockdowns (RD = 0.21, 95% CI = -0.04 to 0.47, *I^2^* = 96.4%, *tau^2^* = 0.049 for moderate-to-severe anxiety and RD = 0.15, 95% CI = 0.07-0.24, *I^2^* = 68.6%, *tau^2^* = 0.005 for moderate-to-severe depression, respectively) (**Figure 4**, panel B, subpanel a and subpanel b). When comparing only studies comparing mental health outcomes between baseline before outbreaks (T1a) and during a lockdown, we found more significant evidence: RD = 0.32, 95% CI = 0.09-0.55, *I^2^* = 95.5%, *tau^2^* = 0.026 for moderate-to-severe anxiety and RD = 0.18, 95% CI = 0.13-0.23, *I^2^* = 0.0%, *tau^2^* = 0 for moderate-to-severe depression, respectively.

After the lifting of the lockdown, two studies [11,16] reported a decrease in the severity of mental health outcomes compared to pre-lockdown levels. However, Huang et al. (2022) noted fluctuations in depressive symptoms across three phases: 7.6% during the lockdown, 7.2% during the remission period, and 8.1% during the normal prevention period [19].

#### Online learning

Three studies focused on changes in mental health outcomes during the transition to online learning [17,20,21]. Two studies examined the transition of students from on-campus teaching to a fully online teaching model [17,21]. These studies reported a significant deterioration in anxiety between the beginning and end of the semester but before final examinations (MD = 2.42, SD = 13.32, *P* < 0.001) [21]. However, when considering all aspects of online learning, study and assessments, the anxiety level had improved at the end of the semester (odds ratio (OR) = -0.10, 95% CI = -0.13 to -0.07). In a study by Schinder et al. (2021), no change in depression was found before and after online learning (*P* > 0.99), but when comparing the baseline, measured in October 2019 before the COVID-19 outbreak, with the online learning period, there was a significant deterioration in depression (October 19 vs. June 2020 and October 2019 vs. December 2020, all *P* < 0.01). In a study conducted by Seffrin et al. (2022), it was noted that students underwent a period without any in-person or online classes before the online learning period. The study found a significantly lower proportion of students reporting moderate-to-severe depression symptoms after the return to the online classes (60.0% before and 52.5% after online learning, *P* = 0.02). However, this change was not observed for moderate-to-severe anxiety status, which remained at 47.5% both before and after the transition to online learning (*P* = 0.61) [20].

## DISCUSSION

This review found evidence that worse mental health outcomes, especially depression among university students, were observed during the implementation of COVID-19 mitigation measures, compared to pre-mitigation periods. The proportion of respondents reporting moderate-to-severe anxiety and depression rose during periods when COVID-19 mitigation measures were in place, with depression showing a particularly noticeable increase. Of note, there was considerable heterogeneity in both baseline and subsequent prevalence estimates, reflecting the diversity of contexts and populations, COVID-19 mitigation measures, the stage of the pandemic locally and measurement instruments employed by the studies. The direction of these effects aligns with findings from two surveys run in the UK in November 2020 and a recent systematic review by Sun et al. (2023). A survey of undergraduate students conducted by the Higher Education Policy Institute (HEPI) found that 58% of respondents reported that their mental health had deteriorated because of the pandemic, compared to 14% who reported an improvement [27]. The Coronavirus Student Survey Phase 3, conducted by the National Union of Students, found that 52% of respondents reported a deterioration in their mental health during the COVID-19 period, compared to 8% who reported an improvement [28]. However, the University College London COVID-19 Social Study found that these declines were not unique to students, but similar to the general population of 18- to 29-year-olds [29]. A systematic review by Sun et al. (2023) found no significant changes in anxiety symptoms (SMD = -0.07, 95% CI = -0.21 to 0.06, *I^2^* = 96%, 16 studies) but a statistically significant worsening in depressive symptoms (SMD = 0.14, 95% CI = 0.01-0.26, *I^2^* = 98%, 19 studies) during the COVID-19 pandemic [30]. Although these studies provided valuable findings, they did not explicitly investigate the impact of COVID-19 mitigation measures. Instead, they compared mental health outcomes in cohorts before and during the pandemic. In contrast, our study strengthened these associations by focusing on COVID-19 mitigation measures and comparing mental health outcomes at different baseline time points. This approach provides a clearer understanding of how these measures impact mental health.

Most participants included in our review were women / females, which may have influenced the changes in mental health outcomes observed. The review by Sun et al. (2023) noted increased levels of anxiety and depression among female participants (SMD = 0.20, 95% CI = 0.12-0.29, *I^2^* = 41% for anxiety; and SMD = 0.22, 95% CI = 0.05-0.40, *I^2^* = 89% for depression) compared with male participants (SMD = 0.07, 95% CI = -0.01 to 0.14, *I^2^* = 0% for anxiety; SMD = 0.01, 95% CI = -0.14 to 0.16, *I^2^* = 82% for depression) [30]. Similarly, a systematic review by Dal Santo et al. (2022) found similar results showing worsening anxiety and depression symptoms in women / females than men / males (SMD = 0.15, 95% CI = 0.07-0.22, *I^2^* = 3.0% for anxiety; SMD = 0.12, 95% CI = -0.09 to 0.33, *I^2^* = 69.0% for depression, respectively) [31]. Although identifying potential risk or protective factors affecting mental health outcomes by gender was challenging in our review, our findings added evidence to highlight the necessity for interventions or support systems tailored to the different needs of the student population.

While most studies included in our analyses focused on lockdown, it was not feasible to compare the intensity or effectiveness of a COVID-19 mitigation measure across different countries. The stringency or duration of a mitigation measure varied by country, depending largely on the preparedness of their health care systems or government readiness for dealing with infectious diseases [32,33]. Furthermore, it was challenging to isolate the effects of a single mitigation measure from others. For instance, the transition to online learning often coincided with university closure, which in turn was a direct result of the lockdown. In this case, we classified mitigations based on the focus of the study. Hence, our analyses offer a general perspective on how mitigation measures have impacted mental health.

The importance of loneliness as a risk factor was echoed by several studies. Feeling extremely isolated, compared to not isolated at all, was significantly associated with depression and anxiety symptom severity and greater odds of moderate anxiety and depression onset. A study by Fruehwirth et al. (2021) found that students who reported feeling usually or always socially isolated mid-pandemic but had not reported social isolation pre-pandemic had a 17.7% increase in depressive symptoms, with effects markedly higher in those with pre-existing depression symptoms [15]. Yang et al. (2021) found that boredom, emotional loneliness, and social loneliness were positively associated with depressive symptoms during the COVID-19 period, although quarantine and lockdown were not significantly associated with depressive symptoms [18]. Weber et al. (2022) echoed that during the lockdown phase, participants with ongoing or deteriorating symptom trajectories exhibited elevated levels of loneliness. Conversely, the most significant increases in loneliness were predominantly seen in those who demonstrated no symptoms of depression and anxiety [22]. These findings are in line with findings from a UK survey conducted in late 2020 [34]. This found that the proportion of students feeling lonely daily or weekly and not feeling part of the university community had increased substantially between May 2019 and October 2020. This was particularly the case for students living in university halls.

Furthermore, other risk factors could also contribute to worsening mental health outcomes in students. The UK-based study by Evans and colleagues did not find increased levels of loneliness compared to the pre-lockdown period [14]. Respondents reported good adaptation to COVID-19 restrictions. The authors did, however, detect high levels of COVID-19-related worry, both for participants themselves and for their relatives. Evans et al. hypothesise that it might be COVID-related worry, rather than the social restrictions related to lockdown, that contribute to the higher rates of depression that they observed during lockdown compared with pre-lockdown [14]. Fruehwirth et al. (2021) and Li et al. (2020) also investigated COVID-related worry or fear, with both studies identifying this as a risk factor for anxiety and depression [9,15]. Coughenour et al. (2021) and Lu et al. (2021) found that low levels of physical activity were a risk factor for both anxiety and depression in students [12,16]. Increased use of mobile gadgets or social media was also identified as a risk factor [10,16,18]. It is plausible that COVID-19 mitigation measures could lead to reduced physical activity, potentially resulting in poor physical health. Moreover, those in poor physical health may express more concerns about COVID-19. The decrease in physical activity could be correlated with increased use of mobile devices or social media, which many adopted as coping mechanisms during the pandemic [35]. The frequent, or excessive, use of smartphones or social media is a concern due to the potential risk to mental health or well-being [35-38].

Our review has several strengths. We conducted an exhaustive search without language or geographic region restrictions and a rigorous article screening and quality assessment. Our review provided more precisely the impact of COVID-19 mitigation measures by examining within-subject comparisons. We analysed both continuous and binary outcomes separately to accommodate a wide range of report types, thereby offering a more comprehensive representation of how mental health outcomes shifted between the two distinct time points. Furthermore, by differentiating baseline time points, we were able to mitigate potential bias that might be caused by differing levels of awareness of COVID-19. This allowed us to provide more reliable evidence when comparing changes in mental health outcomes.

Nonetheless, our review also has several limitations. First, a key limitation of the literature as a whole was that all the studies were opportunistic: all were either new studies, rapid adaptations of existing cohorts or repeat cross-sectional surveys, established quickly in the context of a rapidly developing pandemic. As such, the timing of data collection, the recruitment of participants, and the definition of exposures were sub-optimal. There was a risk of seasonal bias in studies that collected pre- and post-exposure data at different times of the year. Seasonal variation in student well-being is an important factor, which may confound results if data in the exposure and comparator groups are collected at different points in the year. In addition, many of the included studies did not consider wider risk factors for mental health, such as socioeconomic status, living conditions, or external / social support; hence we were unable to identify specific groups in which mental health may have particularly deteriorated. Second, the power of our meta-analyses was notably constrained by differing statistical estimates or the lack of available data. Therefore, future studies such as individual participant data meta-analysis could be an option to resolve high heterogeneity across studies [39]. Although we did not limit our searches by language or geographic regions, most of the included studies were from high-income countries, which could hinder the generalisability of findings to low-middle-income countries (LMICs). Even a large systematic review by COVID-19 Mental Disorders Collaborators, conducted in 2021, did not find any studies published in LMICs [40]. This highlights the urgent need for studies targeting students in low-resource settings to enhance mental health support on campus. Finally, most of the evidence was collected in the early months of the pandemic, thus not addressing the impacts of longer-term and repeated lockdowns on student well-being outcomes.

Our findings have significant implications for universities and for those with responsibility for student welfare. First, institutions must recognise the impact of the pandemic on the level and intensity of depression and anxiety and ensure that robust systems are in place to identify and support students who may be struggling. Second, institutions must also be alert to the fact that some students, such as those suffering from social anxiety or those with pre-existing mental health problems, may have experienced lockdown as a positive escape from the stressors of normal student life. Those responsible for student welfare must be alert to the potential for these students to experience difficulties during the transition back to campus-based learning and again, robust systems should be put in place to ensure early identification and appropriate support. Finally, student welfare and mental health are crucially important, and it is key that both student welfare services and public services are adequately resourced to enable them to support students as we move into a phase of COVID-19 recovery.

## CONCLUSIONS

We reviewed 15 studies examining changes in mental health outcomes amongst university students due to COVID-19 mitigation measures. We found that mental health outcomes, especially depressive symptoms in university students were observed to worsen during COVID-19 mitigations. Governments and institutions should acknowledge the impact of COVID-19 mitigation measures on student anxiety and depression and take appropriate steps to ensure that student welfare and public services are in place to support students effectively.

## Additional material


Online Supplementary Document
