# Supplementary material for: Impact of COVID-19 mitigations on anxiety and depression amongst university students: A systematic review and meta-analysis
Source: J Glob Health. 2023 Sep 1;13:06035. doi: 10.7189/jogh.13.06035 (PMC10472203; doi:10.7189/jogh.13.06035)
Supplement: Online Supplementary Document [file jogh-13-06035-s001.pdf]

Table S1: Search strategy

Search date: 15/02/2023

Database	No	Search terms	Result
**MEDLINE (Ovid)**			
	1	COVID-19/ or SARS-CoV-2/	212344
	2	(2019nCoV or CoV2 or COVID or COVID19* or COVID-19* or COVID-2019* or HCoV-19 or nCoV or SARS2 or SARS?CoV or SARS-CoV or SARS-CoV-2or SARS-CoV-2019 or pandemic).tw,kf.	337383
	3	1 or 2	345510
	4	exp communicable disease control/ or mass screening/ or population surveillance/ or exp masks/ or exp n95 respirators/	584768
	5	(((online or blended or hybrid or mobile or distan* or technology? Mediated or web-enhanced or mix* or asynchronous or synchronous) adj2 (class or lecture* or learning* or education* or learning*)) or e?learning or smart?phone).mp.	38624
	6	(personal protective equipment or facial mask* or face covering* or PPE or N95 or contact trac* or symptom tracking* or ((social or physical) adj2 (distanc* or isolat*)) or lockdown or quarantine*).mp.	85664
	7	("stay at home" or "travel restriction*" or ((external* or international* or oversea* or abroad* or domestic*) adj3 (clos* or closure* or ban or restrict*))).mp.	5512
	8	((school* or colleg* or post-secondary or vocational or technolog* or campus* or universit* or dormitor* or residence* or sororit* or fraternit*) adj2 (clos* or restric*)).mp.	3460
	9	(mitigat* or ventilat* or infecti* preven* or infect* control or testing or test or lateral flow or hand wash*or sanitiz* or sanitis* or clean* or hand hygiene).mp.	2908059
	10	or/4-9	3455181
	11	exp Anxiety/ or exp Anxiety Disorders/	183827
	12	Depression/	146784
	13	(anxiet* or depress*).mp.	778314
	14	11 or 12 or 13	810910
	15	young adult/ or exp students/	1137314
	16	Universities/	51217
	17	15 and 16	26153
	18	(((college* or post-secondary or tertiary or vocational or technical) adj2 (educat* or school* or campus* or universit* or dormitor* or residence* or sorority* or fraternit*)) or graduate* or Undergraduate* or Postgraduate* or Master* or Doctorat* or Advanced education).mp.	254890
	19	17 or 18	273604
	20	3 and 10 and 14 and 19	492
**EMBASE (Ovid)**			
	1	exp coronavirus disease 2019/	314799
	2	(2019nCoV or CoV2 or COVID or COVID19* or COVID-19* or COVID-2019* or HCoV-19 or nCoV or SARS2 or SARS?CoV or SARS-CoV or SARS-CoV-2 or "SARS CoV 2" or SARS-CoV-2019 or pandemic).tw,kf.	414101
	3	1 or 2	437752
	4	exp communicable disease control/ or exp mandatory testing/ or exp social distancing/ or exp mass screening/ or population surveillance/ or mask/	465603
	5	(((online or blended or hybrid or mobile or distan* or technology? Mediated or web-enhanced or mix* or asynchronous or synchronous) adj2 (class or lecture* or learning* or education* or learning*)) or e?learning or smart?phone).mp.	51900
	6	(personal protective equipment or facial mask* or face covering* or PPE or N95 or contact trac* or symptom tracking* or ((social or physical) adj2 (distanc* or isolat*)) or lockdown or quarantine*).mp.	112940
	7	("stay at home" or "travel restriction*" or ((external* or international* or oversea* or abroad* or domestic*) adj3 (clos* or closure* or ban or restrict*))).mp.	7450

	8	((school* or colleg* or post-secondary or vocational or technolog* or campus* or universit* or dormitor* or residence* or sororit* or fraternit*) adj2 (clos* or restric*)).mp.	4020
	9	(mitigat* or ventilat* or infecti* preven* or infect* control or testing or test or lateral flow or hand wash*or sanitiz* or sanitis* or clean* or hand hygiene).mp.	5088323
	10	or/4-9	5413066
	11	exp depression/ or exp anxiety/ or exp anxiety assessment/ or exp depression assessment/	797552
	12	(anxiet* or depress*).mp.	1151294
	13	11 or 12	1198434
	14	exp university student/	14444
	15	(((college* or post-secondary or tertiary or vocational or technical) adj2 (educat* or school* or campus* or universit* or dormitor* or residence* or sorority* or fraternit*)) or graduate* or Undergraduate* or Postgraduate* or Master* or Doctorat* or Advanced education).mp.	297940
	16	14 or 15	309752
	17	3 and 10 and 13 and 16	778
**APA PsycInfo (Ovid)**			
	1	exp covid-19/	17246
	2	(2019nCoV or CoV2 or COVID or COVID19* or COVID-19* or COVID-2019* or HCoV-19 or nCoV or SARS2 or SARS?CoV or SARS-CoV or SARS-CoV-2 or SARS-CoV-2019 or pandemic).tw,hw.	30531
	3	1 or 2	30531
	4	exp disease surveillance/ or exp health screening/ or exp personal protective equipment/ or exp Preventive Health Behavior/ or exp Physical Distancing/	18803
	5	(((online or blended or hybrid or mobile or distan* or technology? Mediated or web-enhanced or mix* or asynchronous or synchronous) adj2 (class or lecture* or learning* or education* or learning*)) or e?learning or smart?phone).mp.	24827
	6	(personal protective equipment or facial mask* or face covering* or PPE or N95 or contact trac* or symptom tracking* or ((social or physical) adj2 (distanc* or isolat*)) or lockdown or quarantine*).mp.	31838
	7	("stay at home" or "travel restriction*" or ((external* or international* or oversea* or abroad* or domestic*) adj3 (clos* or closure* or ban or restrict*))).mp.	1528
	8	((school* or colleg* or post-secondary or vocational or technolog* or campus* or universit* or dormitor* or residence* or sororit* or fraternit*) adj2 (clos* or restric*)).mp.	1895
	9	(mitigat* or ventilat* or infecti* preven* or infect* control or testing or test or lateral flow or hand wash*or sanitiz* or sanitis* or clean* or hand hygiene).mp.	847329
	10	4 or 5 or 6 or 7 or 8 or 9	903250
	11	exp Anxiety Disorders/ or exp Anxiety/	136770
	12	exp "Depression (Emotion)"/	26817
	13	(anxiet* or depress*).mp.	569417
	14	11 or 12 or 13	585651
	15	exp College Students/	102583
	16	(((college* or post-secondary or tertiary or vocational or technical) adj2 (educat* or school* or campus* or universit* or dormitor* or residence* or sorority* or fraternit*)) or graduate* or Undergraduate* or Postgraduate* or Master* or Doctorat* or Advanced education).mp.	256403
	17	15 or 16	320086
	18	3 and 10 and 14 and 17	282
**CINAHL (EBSCO)**			
Expanders - Apply equivalent subjects Search modes - Find all my search terms	1	MH ("COVID-19" OR "SARS-CoV-2" OR "COVID-19 Pandemic" OR "Coronavirus Infections+" OR "Coronavirus+") OR TI (Corona Virus or Coronavirus * or Coronovirus * or CoV or CoV2 or COVID or COVID19 * or COVID-19 or COVID-2019 or HCoV-19 or nCoV or 2019nCoV or SARS2 or SARSCoV or SARS-CoV or 2019-nCoV or SARS-CoV-2 or SARS CoV 2 or SARS-CoV-2019)	121,135
	2	TX ((((college* or post-secondary or tertiary or vocational or technical) N2 (educat* or school* or campus* or universit* or	614,311

		dormitor* or residence* or sorority* or fraternit*)) or graduate* or Undergraduate* or Postgraduate* or Master* or Doctorat* or Advanced education))	
	3	TX (or anxi* OR depress*)	305,669
	4	(MH "Online Education") OR (MH "Education, Non-Traditional") OR "online learning or e-learning or distance learning or online courses or virtual courses or distance education or online education" OR TX (online learning or e-learning or distance learning) OR TX ((((online or blended or hybrid or mobile or distan* or technology? Mediated or web-enhanced or mix* or asynchronous or synchronous) N2 (class or lecture* or learning* or education* or learning*)) or e?learning or smart?phone))	37,320
	5	(MH "Personal Protective Equipment+") OR "personal protective equipment or ppe or gloves or mask or gowns" OR TX (personal protective equipment or facial mask* or face covering* or PPE or N95 or contact trac* or symptom tracking* or ((social or physical) N2 (distanc* or isolat*)) or lockdown or quarantine*)	47,486
	6	TX ("stay at home" or ((external* or international* or oversea* or abroad* or domestic*) N3 (clos* or closure* or ban or restrict*))))	5,831
	7	TX((school* or colleg* or post-secondary or vocational or technolog* or campus* or universit* or dormitor* or residence* or sororit* or fraternit*) N2 (clos* or restric*))	2,338
	8	(MH "Infection Control+") OR "infection control" OR TX (mitigat* or ventilat* or infecti* preven* or infect* control or testing or test or lateral flow or hand wash*or sanitiz* or sanitis* or clean* or hand hygiene)	1,500,776
	9	S4 OR S5 OR S6 OR S7 OR S8	1,560,801
	10	S1 AND S2 AND S3 AND S9	630
**WHO COVID-19 Database https://search.bvsalud.org/global-literature-on-novel-coronavirus-2019-ncov/**			
	1	((((online OR blended OR hybrid OR mobile OR distan* OR technology? mediated OR web-enhanced OR mix* OR asynchronous OR synchronous) AND (class OR lecture* OR learning* OR education* OR learning*)) OR e?learning OR smart?phone)<br>OR<br>(personal protective equipment OR facial mask* OR face covering* OR ppe OR n95 OR contact trac* OR symptom tracking* OR ((social OR physical) AND (distanc* OR isolat*)) OR lockdown OR quarantine*)<br>OR<br>("stay at home" OR ((external* OR international* OR oversea* OR abroad* OR domestic*) AND (clos* OR ban OR restrict*)))<br>OR<br>((school* OR colleg* OR post-secondary OR vocational OR technolog* OR campus* OR universit* OR dormitor* OR residence* OR sororit* OR fraternit*) AND (clos* OR restric*))<br>OR<br>(mitigat* OR ventilat* OR infecti* preven* OR infect* control OR testing OR test OR lateral flow OR hand wash*or sanitiz* OR sanitis* OR clean* OR hand hygiene))<br>**AND**<br>(anxi* OR depress*)<br>**AND**<br>((((college* OR post?secondary OR tertiary OR vocational OR technical) AND (educat* OR school* OR campus* OR universit* OR dormitor* OR residence* OR sorority* OR fraternit*)) OR graduate* OR undergraduate* OR postgraduate* OR master* OR doctorat* OR advanced education))	2707
**ERIC (EBSCO)**			
Expanders - Apply equivalent subjects Search modes - Find all my search terms	1	TX ("COVID-19" OR "SARS-CoV-2" OR "COVID-19 Pandemic" OR "Coronavirus Infections+" OR "Coronavirus+" OR Corona Virus or Coronavirus * or Coronovirus * or CoV or CoV2 or COVID or COVID19 * or COVID-19 or COVID-2019 or HCoV-19 or nCoV or 2019nCoV or SARS2 or SARSCoV or SARS-CoV or 2019-nCoV or SARS-CoV-2 or SARS CoV 2 or SARS-CoV-2019)	11,936
	2	TX (((college* or post?secondary or tertiary or vocational or technical) N2 (educ* or school* or campus* or universit* or dormitor* or residence* or sorority* or fraternit*)) or Graduate* or Undergraduate* or Postgraduate* or Master* or Doctorat* or Advanced education)	337,617

	3	TX anxi* OR depress*	37,047
	4	(TX (((online or blended or hybrid or mobile or distan* or technology? Mediated or web-enhanced or mix* or asynchronous or synchronous) N2 (class or lecture* or learning* or education* or learning*)) or e?learning or smart?phone))	53,677
	5	(TX (personal protective equipment or facial mask* or face covering* or PPE or N95 or contact trac* or symptom tracking* or ((social or physical) N2 (distanc* or isolat*)) or lockdown or quarantine*))	6,043
	6	(TX ("stay at home" or ((external* or international* or oversea* or abroad* or domestic*) N3 (clos* or closure* or ban or restrict*))))	408
	7	(TX((school* or colleg* or post-secondary or vocational or technolog* or campus* or universit* or dormitor* or residence* or sororit* or fraternit*) N2 (clos* or restric*)))	7,687
	8	(TX (mitigat* or ventilat* or infecti* preven* or infect* control or testing or test or lateral flow or hand wash*or sanitiz* or sanitis* or clean* or hand hygiene)	304,162
	9	S4 OR S5 OR S6 OR S7 OR S8	357,423
	10	S1 AND S2 AND S3 AND S9	104
**SCOPUS**			
	1	TITLE-ABS-KEY (2019ncov OR "corona virus*" OR coronavirus* OR coronovirus* OR cov OR cov2 OR covid OR covid19* OR covid-19* OR covid-2019* OR hcov-19 OR ncov OR sars2 OR sarscov OR sars-cov OR 2019-ncov-19 OR sars-cov-2 OR "sars cov 2" OR sars-cov-2019)	503,719
	2	TITLE-ABS-KEY ((((online OR blended OR hybrid OR mobile OR distan* OR web-enhanced OR mix* OR ?synchronous) W/2 (class* OR lecture* OR learning* OR education*)) OR e-learning OR smartphone*))	344,859
	3	TITLE-ABS-KEY ((("personal protective equipment*" OR "fac* mask*" OR "face covering*" OR ppe OR n95 OR "contact trac*" OR "symptom tracking*" OR ((social OR physical) W/2 (distanc* OR isolat*)) OR lockdown OR quarantine* OR mitigation*)))	371,656
	4	TITLE-ABS-KEY (("stay at home" OR ((external* OR international* OR oversea* OR abroad* OR domestic* OR travel*) W/2 (clos* OR closure* OR ban OR restrict*))))	16,493
	5	TITLE-ABS-KEY (((school* OR colleg* OR post-secondary OR vocational OR technolog* OR campus* OR universit* OR dormitor* OR residence* OR sororit* OR fraternit*) W/2 (clos* OR restric*)))	16,869
	6	TITLE-ABS-KEY ((mitigat* OR ventilat* OR "infecti* preven*" OR "infect* control" OR test* OR "lateral flow" OR "hand wash*" OR sanitiz* OR sanitis* OR clean* OR "hand hygiene"))	12,339,615
	7	2 OR 3 OR 4 OR 5 OR 6	12,760,617
	8	TITLE-ABS-KEY ((anxi* OR anxiet* OR depress*))	1,323,510
	9	TITLE-ABS-KEY ((((college* OR post-secondary OR tertiary OR vocational OR technical) W/2 (educat* OR school* OR campus* OR universit* OR dormitor* OR residence* OR sorority* OR fraternit*)) OR graduate* OR undergraduate* OR postgraduate* OR master* OR doctorat* OR advanced*))	2,119,106
	10	1 AND 7 AND 8 AND 9	918
**Science Citation Index (Web of Science)**			
	1	TS=((((2019nCoV or CoV2 or COVID or COVID19* or COVID-19* or COVID-2019* or HCoV-19 or nCoV or SARS2 or SARS?CoV or SARS-CoV or SARS-CoV-2 or SARS-CoV-2019 or pandemic)))) Editions: WOS.SCI	311,187
	2	ALL=((((online or blended or hybrid or mobile or distan* or technology? Mediated or web-enhanced or mix* or asynchronous or synchronous) AND (class or lecture* or learning* or education* or learning*)) or e?learning or smart?phone)) Editions: WOS.SCI	594,697
	3	ALL=((personal protective equipment or facial mask* or face covering* or PPE or N95 or contact trac* or symptom tracking* or ((social or physical) AND (distanc* or isolat*)) or lockdown or quarantine*)) Editions: WOS.SCI	434,386
	4	ALL=(("stay at home" or ((external* or international* or oversea* or abroad* or domestic*) AND (clos* or closure* or ban or restrict*)))) Editions: WOS.SCI	338,964
	5	ALL=(((school* or colleg* or post-secondary or vocational or technolog* or campus* or universit* or dormitor* or residence* or sororit* or fraternit*) AND (clos* or restric*))) Editions: WOS.SCI	1,528,061

	6	ALL=((mitigat* or ventilat* or infecti* preven* or infect* control or testing or test or lateral flow or hand wash*or sanitiz* or sanitis* or clean* or hand hygiene)) Editions: WOS.SCI	6,403,219
	7	#6 OR #5 OR #4 OR #3 OR #2 Editions: WOS.SCI	8,519,856
	8	TS=((anxi* or depress*)) Editions: WOS.SCI	716,800
	9	TS=((((college* or post-secondary or tertiary or vocational or technical) AND (educat* or school* or campus* or universit* or dormitor* or residence* or sorority* or fraternit*)) or graduate* or Undergraduate* or Postgraduate* or Master* or Doctorat* or Advanced education)) Editions: WOS.SCI	300,651
	10	#9 AND #8 AND #7 AND #1 Editions: WOS.SCI	579

Table S2: JBI longitudinal study design quality assessment

Author-Year	Q1	Q2	Q3	Q4	Q5	Q6	Q7	Q8	Q9	Q10	Q11	Scoring	No. of Yes	Overall Appraisal
Elmer 2020	Yes	Yes	Yes	Yes	Yes	No	Yes	Yes	No	No	Yes	11	8	High quality
Li 2020	Yes	Yes	Yes	Yes	Yes	No	Yes	Yes	No	No	Yes	11	8	High quality
Zhang 2020	Yes	Yes	Yes	Yes	Yes	No	Yes	Yes	No	No	Yes	11	8	High quality
Charbonnier 2021	Yes	Yes	Yes	Yes	No	No	Yes	Yes	No	No	Yes	11	7	Moderate quality
Coughenour 2021	Yes	Yes	Yes	Yes	Yes	No	Yes	Yes	No	No	Yes	11	8	Moderate quality
Conceição 2021	Yes	Yes	Yes	No	No	No	Yes	Yes	No	No	Yes	11	6	Moderate quality
Evans 2021	Yes	Yes	Yes	Yes	Yes	No	Yes	Yes	No	No	Yes	11	8	High quality
Fruehwirth 2021	Yes	Yes	Yes	Yes	Yes	No	Yes	Yes	No	No	Yes	11	8	High quality
Lu 2021	Yes	Yes	Yes	Yes	No	No	Yes	Yes	No	No	Yes	11	7	Moderate quality
Schindler 2021	Yes	Yes	Yes	No	No	No	Yes	Yes	No	No	Yes	11	6	Moderate quality
Yang	Yes	Yes	Yes	Yes	Yes	No	Yes	Yes	No	No	Yes	11	8	High quality
Huang 2021	Yes	Yes	Yes	Yes	No	No	Yes	Yes	No	No	No	11	6	Moderate quality
Seffrin 2022	Yes	Yes	Yes	No	No	No	Yes	Yes	No	No	Yes	11	6	Moderate quality
Slykerman 2022	Yes	Yes	Yes	Yes	Yes	No	Yes	Yes	No	No	Yes	11	8	High quality
Weber 2022	Yes	Yes	Yes	No	No	No	Yes	Yes	No	No	Yes	11	6	Moderate quality

Note:

Q1: Were the two groups similar and recruited from the same population?
Q2: Were the exposures measured similarly to assign people to both exposed and unexposed groups?
Q3: Was the exposure measured in a valid and reliable way?
Q4: Were confounding factors identified?
Q5: Were strategies to deal with confounding factors stated?
Q6: Were the groups/participants free of the outcome at the start of the study (or at the moment of exposure)?
Q7: Were the outcomes measured in a valid and reliable way?
Q8: Was the follow up time reported and sufficient to be long enough for outcomes to occur?
Q9: Was follow up complete, and if not, were the reasons to loss to follow up described and explored?
Q10: Were strategies to address incomplete follow up utilized?
Q11: Was appropriate statistical analysis used?

Table S3: Point estimates of outcomes and proportions of clinically significant outcomes amongst university students

Author	Instrument	Mean (SD)			No. of clinical significant cases/total N			
		T1	T2	T3	Cutoff point	T1	T2	T3
**Anxiety**								
Li 2020	PHQ-4	0.95 (0.65)	0.76 (0.61)	NA	NA	NA	NA	NA
Charbonnier 2021	HADS	NA	7.97 (4.14)	6.46 (4.12)	≥11	NA	26/91	14/91
Conceição 2021	GAD-7	NA	NA	NA	≥10	79/366	239/366	NA
Evans 2021	HADS	9.35 (4.28)	9.42 (4.47)	NA	≥8	35/254	87/254	NA
Fruehwirth 2021	GAD-7	5.19 (0.16)	NA	5.41 (0.22)	≥10	76/419	NA	106/419
Lu 2021	GAD-7	NA	NA	NA	≥10	180/5181	NA	190/5181
Seffrin 2022	GAD-7	NA	NA	NA	≥10	19/40	19/40	NA
Slykerman 2022	STAI6	54.31 (12.73)	56.79 (12.67)	NA	NA	NA	NA	NA
Weber 2022	GAD-7	7.50 (4.71)	7.52 (4.30)	NA	≥10	40/135	38/135	NA
**Depression**								
Li 2020	PHQ-4	0.95 (0.65)	0.76 (0.61)	NA	NA	NA	NA	NA
Charbonnier 2021	HADS	NA	6.41 (3.98)	4.23 (3.70)	≥ 8	*NA*	15/91	6/91
Coughenour 2021	PHQ-9	5.58 (4.80)	9.61 (6.91)	NA	NA	NA	NA	NA
Conceição 2021	PHQ-9	NA	NA	NA	≥15	79/366	137/366	NA
Evans 2021	HADS	4.33 (3.26)	6.31 (3.74)		≥ 8	35/254	87/254	NA
Fruehwirth 2021	PHQ-8	6.11 (0.16)	NA	6.23 (0.24)	≥10	90/419	NA	133/419
Lu 2021	PHQ-9	NA	NA	NA	≥10	296/5181	NA	376/5181
Schindler 2021	PHQ-9	T1a (Oct.2019): 5.0 (3.0 - 7.0)* T1b (Dec.2019): 7.0 (4.0 – 11.0)*	T2 (June 2020): 7.0 (5.0 – 10.0)* T2 (Dec.2020) : 7.0 (4.0 – 10.0)*	NA	NA	NA	NA	NA
Yang 2021	CES-D	T1a (Dec.2018): 14.61 (8.35) T1a (June 2019): 15.31 (9.30) T1b (Dec.2019): 15.93 (9.97)	T2 (June 2020): 19.08 (6.63)	NA	≥16	T1a: 81/195 T1a: 89/195 T1b: 94/195	135/195	NA
Huang 2022	PHQ-2	NA	NA	NA	≥3	NA	2700/35516	2877/35516

Author	Instrument	Mean (SD)			No. of clinical significant cases/total N			
		T1	T2	T3	Cutoff point	T1	T2	T3
**Anxiety**								
Seffrin 2022	PHQ-9	NA	NA	NA	≥10	24/40	21/40	NA
Weber 2022	PHQ-8	8.37 (5.52)	8.43 (4.63)	NA	≥10	58/135	58/135	NA

Note: T1a: before COVID-19 measures implementation and before COVID-19 pandemic; T1b: before COVID-19 measures implementation and in early stage of COVID-19 outbreak; T1c: before COVID-19 measures implementation and after COVID-19 pandemic; T2: during COVID-19 measures implementation, T3: after COVID-19 measures implementation, NA: Not available, *Median (Interquartile range, IQR), NA: not available, GAD: Generalized Anxiety Disorder scale, CES-D: Center for Epidemiologic Studies Depression scale, PHQ: Patient Health Questionnaire, HADS: Hospital Anxiety and Depression Scale, STAI6: State Trait Anxiety Inventory 6 item version
